# CARDIOVASCULAR DISEASES PREDICT ALZHEIMER’S DISEASE ONSET IN NON-DEPRESSED ADULTS

**DOI:** 10.1093/geroni/igac059.2746

**Published:** 2022-12-20

**Authors:** Ginny Natale, Sean Clouston

**Affiliations:** Stony Brook University, Stony Brook, New York, United States; Stony Brook University, Stony Brook, New York, United States

## Abstract

We examined the mediating or moderating effect of stroke on the effect of cardiovascular disease (CVD) and the onset of Alzheimer’s disease (AD) in a nationally-representative sample of older Americans with and without clinical depression. Diagnosis of CVD was adjudicated with the established Health and Retirement Study (1992–2016) methodology and included self-reported coronary heart disease, angina, heart failure, myocardial infarction, or other heart conditions. Probable-AD and probable-Stroke were identified inferentially using a validated pattern recognition algorithm. Analyses were stratified by a validated cutoff of the Center for Epidemiologic Studies Depression Scale (CES-D.) Participants (Nf17,154) were observed an average of 8 times over the span of 20 years (Obs=138,510). 12% of all AD cases are preventable by eliminating CVD in older adults in the US, compared to the 31% of all AD cases are preventable by eliminating stroke in the population. In non-depressed adults, risks for accelerated AD onset included: CVD, stroke, age, diabetes, and current smoking. Non-depressed females had delayed AD onset than males. The interaction of CVD x Stroke accelerated AD onset by 1.79 times (95%C.I. =1.63–1.97) in non-depressed adults. CVD was not a significant risk of AD onset in depressed adults. We conclude that 1)reducing the incidence of CVD and strokes in the US would drastically reduce the number of new cases of AD; and 2) stroke compounds the CVD hazards of accelerated AD onset; and 3) clinical depression is a key modulator of the effect of the risk factors of accelerated AD onset.

